# Photoreduction of atrazine from aqueous solution using sulfite/iodide/UV process, degradation, kinetics and by-products pathway

**DOI:** 10.1038/s41598-024-55585-6

**Published:** 2024-03-03

**Authors:** Robabeh Vahidi-Kolur, Ahmadreza Yazdanbakhsh, Seyed Arman Hosseini, Amir Sheikhmohammadi

**Affiliations:** 1 Department of Environmental Health Engineering, School of Public Health and Safety, Shahid Beheshti University of Medical Sciences, Tehran, Iran; 2 Workplace Health Promotion Research Center, Shahid Beheshti University of Medical Sciences, Tehran, Iran; 3 Environmental Health Engineering, Khoy University of Medical Sciences, Khoy, West Azerbaijan Iran

**Keywords:** Chemical engineering, Environmental chemistry

## Abstract

Due to its widespread use in agriculture, atrazine has entered aquatic environments and thus poses potential risks to public health. Therefore, researchers have done many studies to remove it. Advanced reduction process (ARP) is an emerging technology for degrading organic contaminants from aqueous solutions. This study was aimed at evaluating the degradation of atrazine via sulfite/iodide/UV process. The best performance (96% of atrazine degradation) was observed in the neutral pH at 60 min of reaction time, with atrazine concentration of 10 mg/L and concentration of sulfite and iodide of 1 mM. The kinetic study revealed that the removal of atrazine was matched with the pseudo-first-order model. Results have shown that reduction induced by ${{\text{e}}}_{{\text{aq}}}^{-}$ and direct photolysis dominated the degradation of atrazine. The presence of anions (${{\text{Cl}}}^{-}$,${{\text{CO}}}_{3}^{2-}$ and ${{\text{SO}}}_{4}^{2-}$) did not have a significant effect on the degradation efficiency. In optimal conditions, COD and TOC removal efficiency were obtained at 32% and 4%, respectively. Atrazine degradation intermediates were generated by de-chlorination, hydroxylation, de-alkylation, and oxidation reactions. Overall, this research illustrated that Sulfite/iodide/UV process could be a promising approach for atrazine removal and similar contaminants from aqueous solutions.

## Introduction

The excessive use of agricultural chemicals in the world has caused concern about environmental pollution^1,2^. Agricultural chemicals include insecticides, fungicides, and herbicides^3^. Among these substances, atrazine (2-chloro-4-ethylamino-6-isopropylamino-1,3,5-triazine) is a herbicide of s-triazine group, which is widely used to control broad-leaved weeds especially in sugarcane and corn cultivation^4–6^. The physicochemical properties and molecule structure of atrazine are shown in (Table 1)^5,7,8^.

**Table 1:** Physicochemical properties and molecule structure of atrazine.

International union of pure and applied chemistry name (IUPAC name)	2-chloro-4-ethylamino-6-isopropylamino-1,3,5-triazine
Empirical formula	C_8_H_14_ClN_5_
Chemical structure	
Chemical abstract services registry number (CAS no)	1912-24-9
Physical characteristics	Solid and colorless
Solubility in water	34.7 mg/L (22 °C) and 33 mg/L (20 °C)
Density	1.23 g/cm^3^ (22 °C)
Molecular weight	215.68
pK_a_	1.67

The solubility in water (log K_ow_ = 2.6–2.71), long half-life (30–100 days), and long-term use of atrazine lead to the remaining this herbicide in the soil, surface and underground water, which is a threat to the aquatic ecosystem and public health^9,10^. Atrazine is potentially carcinogenic to humans and causes disruption of endocrine glands, reproductive system, delay in puberty and thyroid lesions^4,10–12^. Nowadays, due to health concerns, the use of atrazine has been banned in some European countries, and it is listed as 76 priority and harmful substances in the European Union Water Framework Directive^5,10^. Thus, it is essential to eliminate atrazine from the aquatic ecosystem. Different methods for atrazine removal have been used such as physical^13^, chemical^10,14^, biological techniques^7^ and combined processes^15^. However, these techniques often have limitations. For example, physical methods such as adsorption do not completely destroy the pollutant but instead, transfer it from one phase to another^15^. Therefore, promising technologies are needed to remove toxic pollutants from water bodies. Today, a new technology called advanced reduction processes (ARPs) has been developed alongside advanced oxidation processes )AOPs)^16,17^. ARPs have proven to be very successful in degrading various pollutants such as acid yellow 17 dye^18^,vinyl chloride^19^, bromate^20^, perchlorate^21^, diclofenac^22^, 1,2-dichloroethane^23^, pyridine^24^, 2, 4, 6-trichlorophenol^25^ and hexavalent chromium^23^. ARPs, with a combination of activation methods and reducing agents, lead to the production of highly reactive free radicals that can decompose pollutants^16,26^. Since partial removal efficiency is observed when using reducing agents alone, appropriate activation methods are required to improve its efficiency^16^. Among the different activation methods, we can mention UV-L, UV-N, electron beam, ultrasonic and microwave^16,26^.

The production of ${{\text{e}}}_{{\text{aq}}}^{-}$ in environmental conditions is the action basis of ARPs^27^. Hydrated electron is one of the most active species with standard reduction (− 2.9 V), and it can be produced through various mechanisms including sulfite/UV radiation, sulfite/iodide/UV radiation^21–23,27,28^. UV radiation by activating $\it \it {{\text{SO}}}_{3}^{2-}\mathrm{and H}{{\text{SO}}}_{3}^{-}$ causes the production ${{\text{e}}}_{{\text{aq}}}^{-}$, ${{\text{SO}}}_{3}^{\cdot -}\mathrm{ and }{{\text{H}}}^{\cdot }$ according to Eqs. (1) and (2)^16,22^.

1 $${{\text{SO}}}_{3}^{2-}+{\text{hv}}\to {{\text{SO}}}_{3}^{\cdot -}+{\mathrm{ e}}_{{\text{aq}}}^{-},$$

2 $${{\text{HSO}}}_{3}^{-}+{\text{hv}}\to {{\text{SO}}}_{3}^{\cdot -}+{{\text{H}}}^{\cdot }.$$

But the main limitation of the sulfite/UV process is the need for high sulfite concentration and high pH, also the low efficiency of producing ${{\text{e}}}_{{\text{aq}}}^{-}$^29^. The activation of iodine and its various compounds (e.g. I^–^, I_2_, and IO_4_^–^) has caused the production of reactive species such as ${{\text{e}}}_{{\text{aq}}}^{-}$, ${{\text{IO}}}_{3}^{\cdot }$, ^1^O_2_ and ${{\text{I}}}^{\cdot }$, which are effective in removing pollutants and this has caused researchers to pay attention to this substance^29^. The use of this process for the removal of refractory pollutants have been successful, but the efficiency of the reduction process is largely affected from dissolved oxygen and accumulated reactive iodine species (e.g. ${{\text{I}}}_{3}^{-}$) that cause rapid inhibition of ${{\text{e}}}_{{\text{aq}}}^{-}$^24,29^. Photolysis of iodide using UV light causes the production of ${{\text{e}}}_{{\text{aq}}}^{-}$ (Eq. 3) and RIS in an aqueous solution. Reactive iodine species (RIS) are scavengers of ${{\text{e}}}_{{\text{aq}}}^{-}$. Therefore, to overcome the disadvantages of the iodine ions produced, a reducing agent such as sulfite is used. The sulfite ions can scavenger by RIS in the reaction and thus prevent the reduction of ${{\text{e}}}_{{\text{aq}}}^{-}$ with RIS^30^.

Researchers have done many studies on atrazine removal by different mechanisms and UV-based processes. However, these studies have the disadvantage of a long reaction time and low percent of degradation atrazine^31–34^. On the other hand, the kinetics and degradation mechanism of atrazine in the presence of two reducing agents (sulfite and iodide ions) and UV irradiation have not been investigated yet. In this study, the sulfite/iodide/UV process is proposed to use the efficient and beneficial effects of sulfite and iodide along with UV to remove atrazine. Because according to this process, UV light is absorbed by iodide and sulfite that both operate as ${{\text{e}}}_{{\text{aq}}}^{-}$ precursors^23,27,29^. ARPs process can react with persistent and halogenated organic pollutants^23,29^.

3 $${{\text{I}}}^{-}+{\text{hv}} \to {{\text{I}}}^{*}-{\text{CTTS}} \to {{\text{I}}}^{\cdot }+{\mathrm{ e}}_{{\text{aq}}}^{-}.$$

Therefore, the objective of this study was to evaluation of atrazine removal by sulfite/iodide/UV process from aqueous solutions. We focused on (a) investigating the effects of various parameters on the removal performance of atrazine (e.g. pH, sulfite and iodide concentration, initial atrazine concentration, scavengers, and effect of UV); (b) studying reaction kinetics; (c) determining the roles of sulfite and iodide; (d) detecting the degree of mineralization of atrazine and (e) determining the by-products.

## Materials and methods

### Chemicals

All chemicals were analytical reagent grade and used without further purification. Potassium iodide (KI; 99.5%), sodium sulfite (Na_2_SO_3_; > 95%), sodium hydroxide (NaOH), hydrochloric acid (HCL), atrazine (2-chloro-4-ethylamino-6-isopropylamino-1,3,5-triazine, purity ≥ 98%) and sodium carbonate (Na_2_CO_3_), sodium sulfate (Na_2_SO_4_), sodium chloride (NaCl), sodium nitrate (NaNO_3_), sodium nitrite (NaNO_2_) and ethanol were purchased from Merck Co., Germany. Methanol (CH_3_OH HPLC grade; 99.9%) and HPLC grade water were purchased from Chem Lab Company, Belgium.

### Photoreactor set up

**Figure 1:**
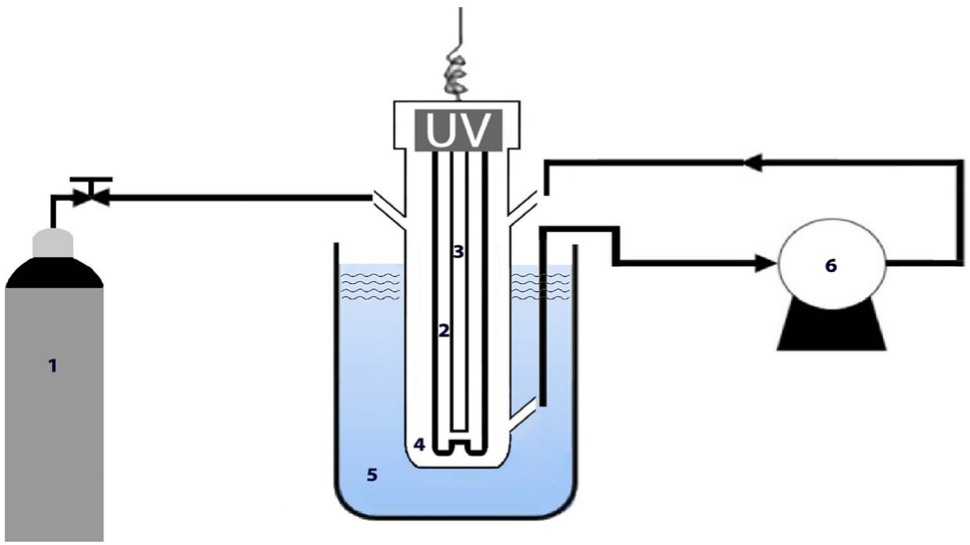
Schematic diagram of the experimental set-up. (1-nitrogen gas, 2-quartz tube, 3-low pressure UV lamp, 4- reactor, 5- cooling water, 6 -diaphragm pump).

The experiments were conducted on a pilot scale. A schematic diagram of the experimental set-up is shown in (Fig. 1). The reactor was a tubular glass of 30 cm in height and 3 cm in diameter. A quartz tube was placed in the center of the reactor. A low-pressure mercury lamp that emitted UV radiation at a wavelength of approx. 254 nm was inserted in the quartz tube. Prior to each stage of the experiment, the UV lamp (Phillips) was switched on for at least 15 min to warm up and reach a stable output of the photon flux. Water fed to the reactor was purged with N_2_ for 10 min before the start of the experiment to remove any dissolved oxygen. The reactor was perforated at 5 cm from the bottom and top to allow the recirculation of water. Mixing in the reactor was carried out by recirculating water through a diaphragm pump was operated. The heating effect of the UV lamp is decreased by cooling water in the beaker around the reactor. Also, during the experiments, the reactor was wrapped with an aluminum sheet to prevent light from entering the reactor and to ensure the safe operation of the system.

### Experimental procedure

In this study, the 30 mg/L stock solution of atrazine was prepared by dissolving 3 mg of atrazine (99.9% purity) in a certain amount of methanol and bringing to volume of 100 mL with ultrapure water. Then, to complete the dissolution of atrazine, the solution was stirred at room temperature for 24 h in the brown-amber glass bottles covered with aluminum foil. In continuation of work, the stock solution of atrazine was passed through a 0.45 μm filter and stored in a refrigerator at 4 °C in the dark to avoid photochemical degradation. Atrazine has limited solubility in water (33 mg/L at 20 °C), and temperature can influence its solubility. So, the solubility of atrazine may decrease at lower temperatures (such as 4 °C), which can lead to the formation of precipitation in the stock solution. To prevent precipitation, the temperature of the stock solution was brought to room temperature before use, and after complete mixing, the working solutions were prepared by diluting the appropriate volume of the stock solution in deionized water. At each run of the experiment, 150 mL of atrazine synthetic solution was injected into the reactor. The initial pH of atrazine stock solution without adjustment was 7.26, and 0.1 N NaOH or HCl was used to adjust the initial pH (3–11). The reactions was continued by the addition of the appropriate amount of iodide and sulfite to the reactor simultaneously. Then, 2 mL of sample were taken out at predetermined times and immediately filtered through 0.45 μm syringe filter discs. Degradation reactions were quenched by adding a certain amount of methanol to the samples before being analyzed by high-performance liquid chromatography (HPLC). The experiments were carried out at room temperature (25 ± 1 °C). In order to study the effects of various parameters, experiments were conducted at different amounts of iodide and sulfite (0 to 4 mM), initial atrazine concentrations (2 to 10 mg/L), initial pH (3 to 11), and time (0 to 80 min). Each degradation experiment was replicated two times.

### Analytical methods

The pH of the solution was measured by a pH meter (Metron, Switzerland). The concentrations of atrazine in the samples were measured by reversed-phase high-performance liquid chromatography (HPLC, KNAUER, Germany) equipped with the Eclipse XDB C-18 (5 μm, 4.6 × 250 mm) column, and the detection was performed using a UV detector at 250 nm. The mobile phase was composed of 20% ultrapure water and 80% methanol, with a flow rate of 1.0 mL/min. The injection volume was 20 μL for each analysis, and the column temperature was set at 25°C. The retention time for atrazine was approximately. 4.88 min. The samples are filtered through 0.45 μm syringe filters (MCE, France).

The degree of atrazine degradation was calculated according to the Eq. (4):

4 $$\mathrm{Degradation\,\, efficiency }(\mathrm{\%}) = \left(\frac{{{\text{C}}}_{0}-{{\text{C}}}_{{\text{t}}}}{{{\text{C}}}_{0}}\right)\times 100,$$

where ${{\text{C}}}_{0}$ and ${{\text{C}}}_{{\text{t}}}$ are the initial and final atrazine concentrations after removal, respectively.

The chemical oxygen demand (COD) was measured by the closed reflux colorimetric analysis in accordance with the Standard Methods for the Examination of Water and Wastewater^35^. The degree of atrazine mineralization (under optimum conditions) was assessed by changes in total organic carbon (TOC) content measured by Shimadzu TOC-L CSN analyzer. Liquid chromatography/mass spectrometry (LC–MS, Micromass Quattro micro API Waters Alliance 2695) analysis was used in order to identify the potential intermediates of atrazine degradation in the process.

### Kinetic study

The decomposition of atrazine was investigated pseudo-first-order and pseudo-second-order kinetic models via Eqs. (5) and (6), respectively**.**

5 $${\text{Ln}}\left(\frac{{{\text{C}}}_{0}}{{{\text{C}}}_{{\text{t}}}}\right)={{\text{k}}}_{{\text{obs}}}\cdot {\text{t}},$$

6 $$\frac{1}{{{\text{C}}}_{0}}-\frac{1}{{{\text{C}}}_{{\text{t}}}}={{\text{K}}}_{2}\cdot {\text{t}},$$

where ${{\text{C}}}_{{\text{t}}}$ and ${{\text{C}}}_{0}$ are the atrazine concentration (mg/L) measured at contact time t and time 0, respectively, t is the reaction time (min), also ${{\text{k}}}_{{\text{obs}}}$,${{\text{k}}}_{2}$ is the observed pseudo-first-order and pseudo-second-order rate constants (${{\text{min}}}^{-1}$), which were be extracted as the slope of ${\text{Ln}}\left( \frac{{{\text{C}}}_{0}}{{{\text{C}}}_{{\text{t}}}}\right)$ and $\frac{1}{{{\text{C}}}_{0}}-\frac{1}{{{\text{C}}}_{{\text{t}}}}$ versus time, respectively.

### The energy consumption

The electrical energy of ARP processes should be considered as a basic parameter in treatment costs. The "electrical energy per order" (E_EO_) for a first-order kinetic model of photodegradation processes explained by Bolton et al. the E_EO_ or EE/O is calculated based on Eqs. (7) and (8).

7 $${\text{EE}}/{\text{O}}=\frac{{\text{P}}\times {\text{t}}\times 1000}{{\text{V}}\times 60\times {\text{log}}\frac{{{\text{C}}}_{0}}{{{\text{C}}}_{{\text{t}}}}},$$

8 $${{\text{E}}}_{{\text{EO}}}=\frac{38.4\times {\text{P}}}{{\text{V}}\times {{\text{K}}}_{{\text{obs}}}},$$

where P, t, V, C_0_, C_t_, k_obs_ and E_EO_ are the power of UV lamp (kW), irradiation time (h), solution volume (m^3^), the initial concentration and final concentration at time t, the pseudo-first-order rate constant (min^−1^) and electrical energy per order (kWh/m^3^), respectively^18,36^.

### Statistical analysis

Statistical analysis of all the data in this study was conducted using Microsoft Excel 2016 and IBM SPSS Statistics 2016.

## Results and discussion

### Effect of pH on atrazine removal

**Figure 2:**
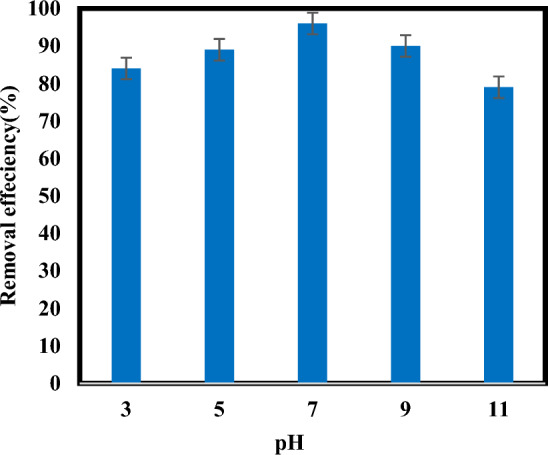
Degradation of atrazine by sulfite/iodide/UV process at different pH values ([${{\text{SO}}}_{3}^{2-}$] and [${{\text{I}}}^{-}$]: ]: 2 mM, atrazine concentration: 10 mg/L, time: 60 min).

The influence of pH on the atrazine degradation by the sulfite/iodide/UV process was investigated. As illustrated in (Fig. 2), the degradation efficiency of atrazine by the sulfite/iodide/UV process were 84%, 89%, 96%, 90% and 79% for pH 3, 5, 7, 9 and 11, respectively. Results showed that sulfite/iodide/UV process performed better in neutral conditions than that in acidic and alkaline conditions and pH = 7 was selected optimum pH.

**Figure 3:**
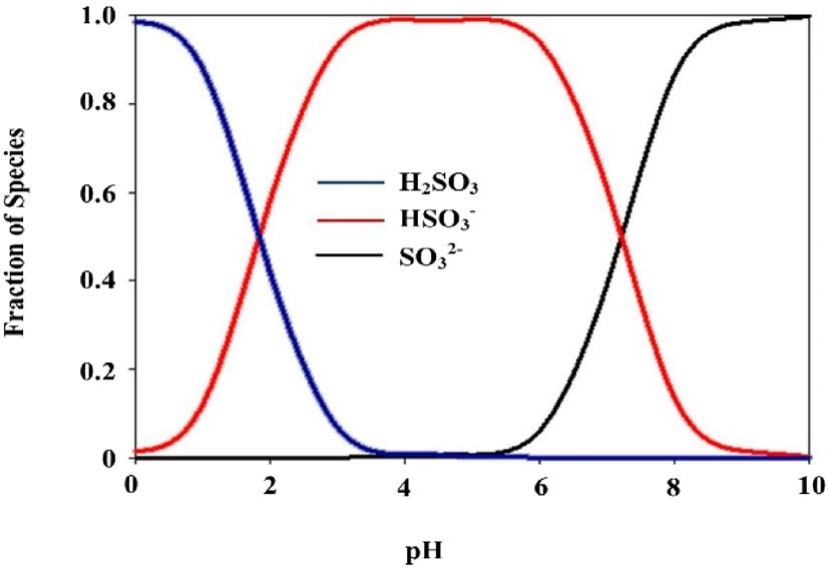
Species distribution of sulfite at various pH values.

The pH of a solution determines the distribution of sulfite species (${{\text{H}}}_{2}{{\text{SO}}}_{3}$, ${{\text{HS}}0}_{3}^{-}$ and ${{\text{SO}}}_{3}^{2-}$) during the degradation process (Fig. 3). That its dominant species are ${{\text{HS}}0}_{3}^{-}$ and ${{\text{SO}}}_{3}^{2-}$ under acidic and alkaline conditions, respectively^37,38^. Due to the difference in the absorption of UV radiation by sulfite dominant species at different pHs, will be produced three reactive specie including ${{\text{e}}}_{{\text{aq}}}^{-}$, ${{\text{SO}}}_{3}^{\cdot -}\mathrm{ and }{{\text{H}}}^{\cdot }$. So that,${{\text{SO}}}_{3}^{2-}$ can absorb significant amounts of UV radiation with wavelength of 254 nm, while ${{\text{HS}}0}_{3}^{-}$ cannot absorb UV radiation in a range of 225–300 nm Therefore, this action shows ${{\text{HS}}0}_{3}^{-}$ does not have enough ability to produce ${{\text{e}}}_{{\text{aq}}}^{-}$ and ${{\text{SO}}}_{3}^{\cdot -}$*,* on the other hand ${{\text{e}}}_{{\text{aq}}}^{-}$ could be scavenged by H^+^ in acidic pH^25,38^. So, pH plays a significant role in conversion between ${{\text{e}}}_{{\text{aq}}}^{-}$ and ${{\text{H}}}^{\cdot }$, through Eqs. (9)–(12)^22,29^.

9 $${{\text{e}}}_{{\text{aq}}}^{-} +{{\text{H}}}_{2}{\text{O}}= {{\text{H}}}^{\cdot }+{{\text{OH}}}^{-}{ ({\text{K}}}_{1 }=1.9\times {10}^{1}{\mathrm{ M}}^{-1}{{\text{S}}}^{-1}),$$

10 $${{\text{e}}}_{{\text{aq}}}^{-}+{{\text{H}}}^{+}={{\text{H}}}^{\cdot } \left({{\text{K}}}_{2 }=2.3\times {10}^{10}{\mathrm{ M}}^{-1}{{\text{S}}}^{-1}\right),$$

11 $${{{\text{e}}}_{{\text{aq}}}^{-}+\mathrm{ HSO}}_{3}^{-}={{\text{H}}}^{+}+ {{\text{SO}}}_{3}^{2-} \left({{\text{K}}}_{3 }=2.0\times {10}^{7}{\mathrm{ M}}^{-1}{{\text{S}}}^{-1}\right),$$

12 $${{\text{H}}}^{\cdot }+{{\text{OH}}}^{-}={\mathrm{ e}}_{{\text{aq}}}^{-}+{{\text{H}}}_{2}\mathrm{O }({{\text{K}}}_{4 }=2.2\times {10}^{7}{\mathrm{ M}}^{-1}{{\text{S}}}^{-1}),$$

In contrast, ${{\text{SO}}}_{3}^{2-}$ has much higher efficiency to generate ${{\text{e}}}_{{\text{aq}}}^{-}$ and free radicals like ${{\text{SO}}}_{3}^{\cdot -}\mathrm{and }{{\text{H}}}^{\cdot }$ in higher pH values (7–11)^25,38^. According to the obtained results, atrazine degradation decreased when increasing the pH from 7 to 11, and the variation was chiefly ascribed to the distribution ratio of sulfite species, the resulting decrease in ${{\text{e}}}_{{\text{aq}}}^{-}$ generation, as well as the consumption of ${{\text{e}}}_{{\text{aq}}}^{-}$ by the competing reactions^16,19,29^. A similar influence of pH on pollutants removal was also observed in ARPs^23,25^. Cong et al.^23^ applied by sulfite/iodide/UV to Cr(VI) removal. Results of their research showed that pH = 7 had a higher efficiency than alkaline pH^23^. Yazdanbakhsh study group (2017) investigated degradation of 2, 4, 6-trichlorophenol by an advanced reduction process based on sulfite anion radical. They indicated that TCP degradation in solution with natural pH is higher than others’ pHs^25^. However, findings of some studies have shown that better removal of pollutants occurred at higher pHs. In research by Botlaguduru et al. for the removal of bromate by UV-sulfite process, the higher removal efficiency was obtained at alkaline pH^38^.

### Effect of contact time on atrazine removal

**Figure 4:**
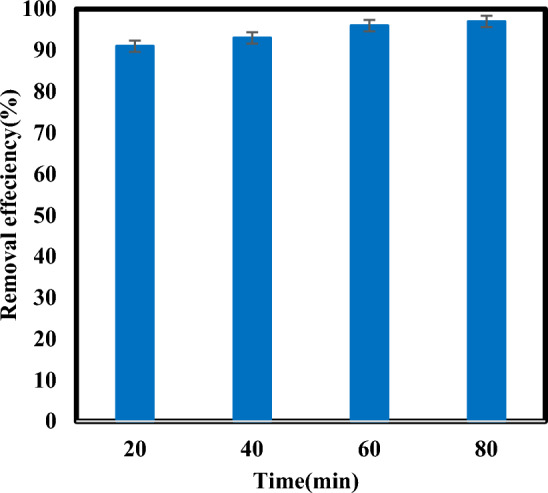
Degradation of atrazine by sulfite/iodide/UV process at different time (pH: 7, [${{\text{SO}}}_{3}^{2-}$] and [${{\text{I}}}^{-}$]: 2 mM, atrazine concentration: 10 mg/L).

The influence of contact time on the removal efficiency of atrazine was investigated at various times. (Fig. 4), shows that the removal efficiency increased from 91 to 97% by increasing the contact time from 20 to 80 min at pH 7. As shown in Fig. 3 not much difference (approximately 1%) was observed in the removal efficiency of 60 between 80 min. therefore, the optimal time for the process was considered 60 min. By increasing the contact time, the conditions for the production of sufficient reduction radicals for degradation are provided and also contact between the active radicals and atrazine is increased^25^. The contact time is important to the economy of the decomposition process. Achieving higher efficiency degradation in less contact time will reduce the energy consumption in the process. Also, achieving the appropriate contact time depends on several factors, such as pollutant concentration, type and amount of chemical used, etc. The degradation of atrazine by sulfite/iodide/UV process with contact time of 20 min also had a high efficiency, which can be economically important and can be investigated. In this study, because all effective parameters have not been examined yet, it is not possible to choose a time of 20 min to continue the work. In the following, after checking the parameters and optimizing them, it was observed that the removal efficiency of atrazine is low in 20 min, and this time cannot be suitable for the optimal time, as well as examining the kinetics of the process. Many studies confirm that increasing the reaction time increases the degradation efficiency. Mousavi et al. increased the removal efficiency of Cr(VI) by increasing the reaction time in real chrome-plating wastewater using a VUV photoreactor^39^.

### Effect of sulfite on atrazine removal

**Figure 5:**
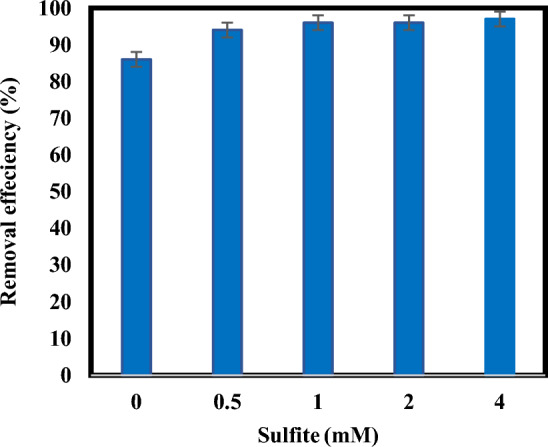
Degradation of atrazine by sulfite/iodide/UV process at different amounts of sulfite (pH: 7, [${{\text{I}}}^{-}$]: 2 mM, atrazine concentration: 10 mg/L, time: 60 min).

Initial sulfite concentration is an important factor in the removal efficiency and the economic costs of the sulfite/iodide/UV process. (Fig. 5), shows that atrazine removal efficiency increased from 86 to 96% by increasing sulfite concentration from 0 to 2 mM, but when sulfite concentration increased from 2 to 4 mM, only 1% enhancement of atrazine degradation was observed. Since the removal efficiency of atrazine was not much different between sulfite concentrations of 1 to 4 mM, the optimal concentration of sulfite was considered to be 1 mM to decrease costs. So further experiments were performed at a concentration of 1 mM of sulfite. At low sulfite concentrations (less than 2 mM), sulfites generate reducing radicals through UV absorption, the number of which is directly related to the initial sulfite concentration. But as the sulfite concentration increases further (from 2 to 4 mM), the sulfite is nearly saturated with light absorption. As a result, the amount of reducing radicals generated by UV/sulfite is also almost saturated and their production will not increase significantly, leading to no appreciable change in the degradation efficiency of atrazine^16,37^. Researchers have proposed two reaction pathways for the removal of pollutants with the ARP process: direct photolysis and reaction with reducing radicals^16,38^. If direct photolysis is the dominant degradation mechanism for atrazine, the average light intensity that may be absorbed by the target contaminant will decrease due to the absorption of a percentage of light by sulfite^40^. If the reaction with reducing radicals is the main degradation mechanism, increasing the amount of sulfite leads to on increase in the target pollutant, which is due to the production of more radicals as a result of more absorption of light by sulfite^16,38^. In this research, the results confirm that the degradation mechanism of atrazine is both free radicals and direct photolysis. Similar results to our work have been reported in studies by other researchers to degradation other compounds^41,42^. Xie study group (2015) investigated enhanced debromination of 4-bromophenol by the UV/sulfite process. They indicated that increasing sulfite concentration higher removal efficiency of 4-bromophenol^43^.

### Effect of iodide on atrazine removal

**Figure 6:**
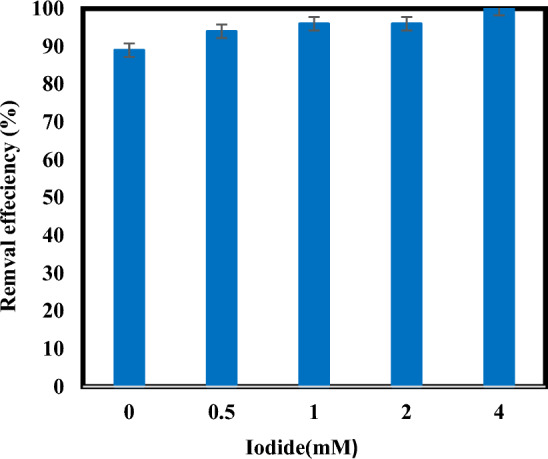
Degradation of atrazine by sulfite/iodide/UV process at different amounts of iodide (pH: 7, [${{\text{SO}}}_{3}^{2-}$]: 1 mM, atrazine concentration: 10 mg/L, time: 60 min).

To determine the effect of iodide concentration, experiments were performed with concentrations of 0 to 4 mM of iodide. As shown in (Fig. 6) the removal efficiency of atrazine at 1 and 2 mM iodide concentrations is similar and equal to 96%. Although 100% removal efficiency has been achieved at the concentration of 4 mM of iodide, from the economic point of view, it is preferable to consider the concentration of 1 mM. The higher removal efficiency of atrazine in the presence of iodide indicates an important role for iodide in degradation. Photolysis of iodide using UV light causes the production of ${{\text{e}}}_{{\text{aq}}}^{-}$ in aqueous solution^44^. On the other hand, iodide absorbs UV light much more effectively than sulfite and also has a higher quantum efficiency, which justifies its greater contribution to the removal of atrazine^23,29^. Therefore, it can be used for the reductive degradation of many resistant pollutants. Cong et al. ^23^ investigated the removal of Cr(VI) at alkaline pHs by sulfite/iodide/UV. In their study, a result similar to our work was observed that by increasing the concentration of iodide from 0 to 0.2, the removal efficiency of Cr(VI) reached from 22 to 99% due to the reason increasing generation of ${{\text{e}}}_{{\text{aq}}}^{-}$ by charge-transfer-to-solvent (CTTS) excitation of iodide is mentioned^23^. The determination of the optimal dosage of iodide and sulfite through one-way experiments could present challenges in fully assessing their synergistic effect on electron production. Also, considering the influence of UV dosage is indeed crucial to understanding the complex dynamics of electron production and subsequent chemical processes. When both iodide and sulfite are concurrently present in a reaction, their combined effect can significantly impact electron production. This synergistic relationship can lead to the generation of a greater quantity of reactive species, potentially influencing the efficacy of degradation processes, such as the photoreduction of contaminants like atrazine. For the above reason, in this study, the simultaneous effects of iodide and sulfite were investigated along with UV. The role of UV radiation in the process is important. The different UV intensities directly affect the extent of photochemical processes, including the excitation of reactive species such as iodide ions and sulfite, thus influencing their ability to generate reactive electrons crucial for degradation reactions^16,25,30^. However, in this study, only UV intensity 87 $\upmu W{cm}^{-2}$ was examined.

### Effect of initial atrazine concentration

**Figure 7:**
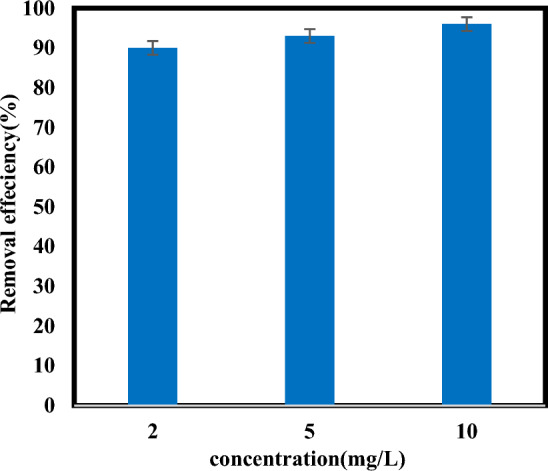
Degradation of atrazine by sulfite/iodide/UV process at different concentrations (pH: 7, [${{\text{SO}}}_{3}^{2-}$] and [${{\text{I}}}^{-}$]: 1 mM, time: 60 min).

The effectiveness of the sulfite/iodide/UV photoreduction process with variations in initial atrazine concentrations ranging from 2 to 10 mg/L is shown in (Fig. 7). The degradation rate was directly correlated with the initial atrazine concentration. Accordingly, the degradation rate at the atrazine concentrations of 2, 5 and 10 mg/L was around 90%, 93% and 96%, respectively. The degradation rate constants of different compounds show different sensitivities changing the initial concentration of the target pollutant^16^. Probably, at higher concentrations, the efficient contact between target pollutant molecules and active species is more, and as a result, their self-consumption decreases and the target pollutant removal efficiency increases^28^. A similar influence of variations in initial concentration on pollutant removal was also observed in researchers' studies^28,45^. Cao and colleagues reported that removal efficiency increased with increasing F–53B initial concentration^28^. However, Yazdanbakhsh et al. presented the opposite result that the removal efficiency of acid yellow 17 dye decreased with increasing initial concentration by the dithionite/UV-C advanced reduction process, which is caused by a decrease in the quantum yield of dithionite ion photolysis into sulfur dioxide radicals^18^.

### Kinetic degradation

**Figure 8:**
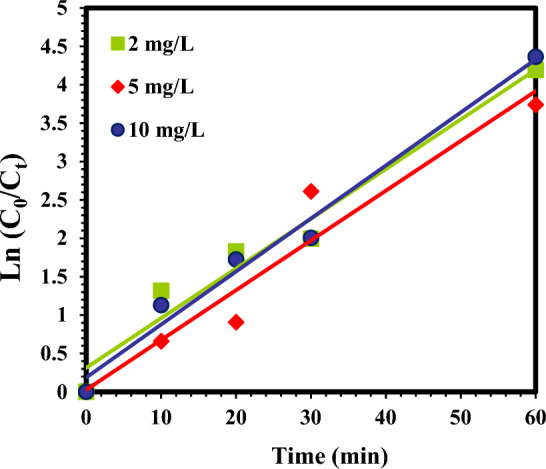
Pseudo-first-order kinetic model atrazine degradation by sulfite/iodide/UV process at different concentrations (pH: 7, [${{\text{SO}}}_{3}^{2-}$] and [${{\text{I}}}^{-}$]: 1 mM, time: 60 min).

**Figure 9:**
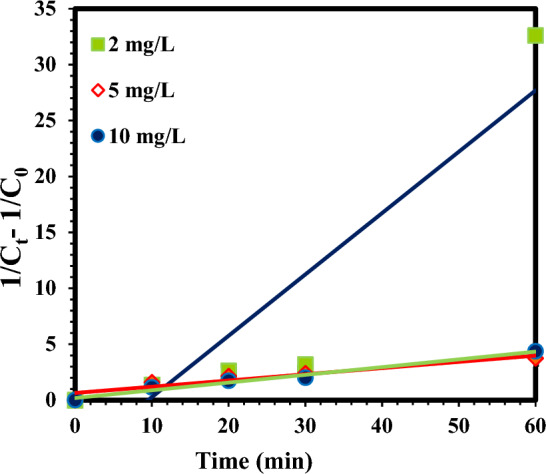
Pseudo-second-order kinetic model atrazine degradation by sulfite/iodide/UV process at different concentrations (pH: 7, [${{\text{SO}}}_{3}^{2-}$] and [${{\text{I}}}^{-}$]1 mM, time: 60 min).

The kinetic analysis was performed to better explain the influence of atrazine concentration on the sulfite/iodide/UV process. The diagrams of kinetics are shown in (Figs. 8 and 9). Also, equations, kinetic coefficients, and R^2^ values for different concentrations of atrazine in the sulfite/iodide/UV process are illustrated in Table 2. In this study, the removal of atrazine at different initial concentrations (R^2^ = 0.9355–0.9818) was matched with pseudo-first-order model. According to kinetic calculations (Table 2), the ${{\text{k}}}_{{\text{obs}}}$ value of atrazine removal was 0.0647, 0.0648 and 0.0961 min^−1^ at concentrations of 2, 5 and 10 mg/L, respectively.

**Table 2:** The calculated kinetic parameters for pseudo-first-order and pseudo-second-order models on atrazine with sulfite/iodide/UV process at different concentrations.

Atrazine concentration (mg/L)	Pseudo-first-order model			E_EO_ (kWh/m^3^)	Pseudo- second order model		
	Equation	${{\text{k}}}_{{\text{obs}}} ({{\text{min}}}^{-1})$	${{\text{R}}}^{2}$		Equation	${\text{K}}$ $({\text{L}}/{{\text{mg}}}^{-1}{{\text{min}}}^{-1})$	${{\text{R}}}^{2}$
2	y = 0.0647x + 0.4119	0.0647	0.9631	47.48068	y = 0.0691x + 0.1866	0.5485	0.9818
5	$\text{y=0.0648x+0.0273}$	0.0648	0.9355	47.4074	$\text{y=0.0557x+0.6388}$	$\text{0.14}$	$\text{0.8929}$
10	y = 0.0961x + 0.1866	0.0961	0.9818	31.9667	y = 0.5485x-5.2159	0.1311	0.8337

### The energy consumption and economic evaluation

As shown in Table 2, the atrazine degradation during the sulfite/iodide/UV process had an electrical energy consumption of 47.48 to 31.96 kWh/m^3^, which was variable with the initial atrazine concentration in the range of 2–10 mg/L. There were inversely correlated k_obs_ with E_EO_, such that with increasing k_obs_, E_EO_ values decreased. Sheikh Mohammadi et al. (2019) reported that in the degradation of trichlorophenol using sulfite anion radicals in a photochemical process combined with a biological reactor, the value of E_EO_ increased with rising TCP concentration^46^. It is suggested according to the obtained results, that due to low electrical energy consumption, the process can be suitable for environmental applications.

### Comparison of atrazine removal efficiency sulfite/iodide/UV, UV/sulfite, sulfite/ iodide and UV alone

**Figure 10:**
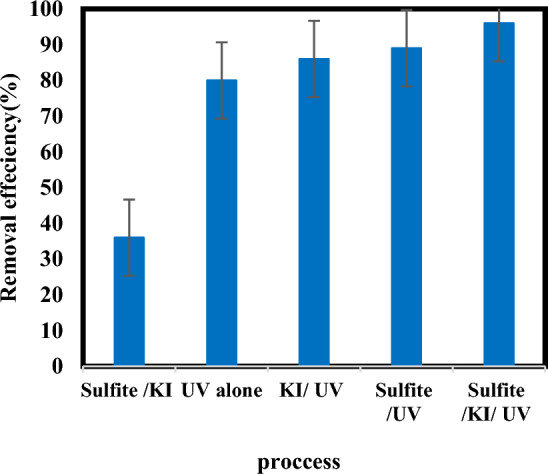
Comparison of atrazine removal efficiency with different processes (pH: 7, [${{\text{SO}}}_{3}^{2-}$] and [${{\text{I}}}^{-}$]: 1 mM, atrazine concentration: 10 mg/L, time: 60 min).

The removal efficiency of atrazine was investigated by five different processes. (Fig. 10) shows that removal of atrazine within 60 min was obtained in the order of sulfite/iodide/UV (96%) > UV/sulfite (89%) > UV/iodide (86%) > UV alone (80%) > sulfite/ iodide (36%). UV in the absence of sulfite and iodide has a significant effect on the removal efficiency of atrazine, which indicates the high contribution of direct photolysis in the degradation of atrazine with this process^43^. Sulfite/iodide process showed low removal efficiency, which could be attributed to the low reduction potential of sulfite/iodide. A higher removal efficiency was observed in UV/sulfite compared to UV/iodide, which indicates more production of ${{\text{e}}}_{{\text{aq}}}^{-}$ in the UV/sulfite process. Probably, the lower ${{\text{e}}}_{{\text{aq}}}^{-}$ concentration in the UV**/**iodide process is because of ${{\text{e}}}_{{\text{aq}}}^{-}$ scavenging reactions of reactive iodine species, like ${{\text{I}}}_{3}^{-}$ and ${{\text{I}}}_{2}^{\cdot -}$, which are generated during photo-oxidation of iodide^23,27,44^. In the sulfite/iodide/UV process, sulfite is activated by UV to produce free radicals that are involved in the further degradation of atrazine^37^. The combination of sulfite and iodide via UV irradiation under anaerobic conditions significantly increased the degradation of atrazine to 96%. This issue is most likely related to the production of active species such as ${{\text{SO}}}_{3}^{\cdot -}$ and ${{\text{e}}}_{{\text{aq}}}^{-}$ by sulfite and iodide decomposition. Results similar to our work have been seen in the research by Zhang et al. (2018) on bromate removal by Iodide-assisted UV/Sulfite process^27^.

According to the results of the comparison experiment, it could be speculated that the contribution of ${{\text{e}}}_{{\text{aq}}}^{-}$ was quite limited (less than 20%). Several factors can cause the limited contribution of ${{\text{e}}}_{{\text{aq}}}^{-}$ in a photoreduction process.

1. In some systems, there may be competing processes that influence the fate of the reactive species.
2. The transformation of atrazine and its by-products involves a network of complex chemical reactions, and other reactive species or pathways may be predominant in the conversion mechanisms, overshadowing the contribution of ${{\text{e}}}_{{\text{aq}}}^{-}$.
3. Factors such as pH, the presence of other organic or inorganic substances, and variations in light intensity can influence the behavior of ${{\text{e}}}_{{\text{aq}}}^{-}$ and their role in the overall degradation process.

The suitability of atrazine degradation with the photoreduction process depends on various factors, such as reaction conditions, type of reactor, reactivity towards the active species produced under UV radiation, etc^16,27,30^. Given that UV radiation contributed up to 80% of atrazine degradation in the sulfite/iodide/UV process, it highlights the potential suitability of atrazine for a photoreduction process. Further investigation could shed light on the interplay of various reactive species and their role in the transformation pathways, offering valuable results into the environmental fate of atrazine and the optimization of the degradation process.

### Effect of scavengers (${{\text{NO}}}_{3}^{-}$, ${{\text{NO}}}_{2}^{-}$, $\mathrm{methanol and ethanol})$

**Figure 11:**
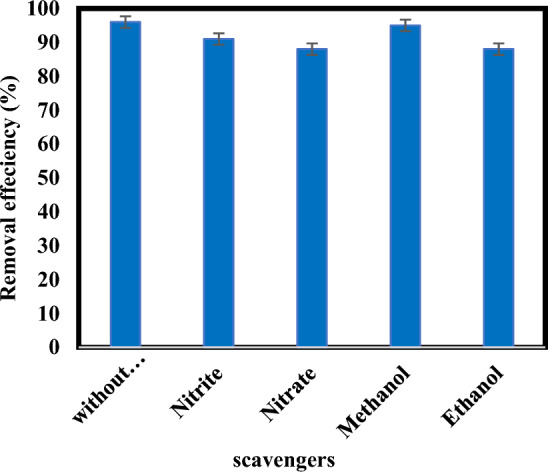
Degradation of atrazine by sulfite/iodide/UV process in presence of scavengers (pH: 7, [${{\text{SO}}}_{3}^{2-}$] and [${{\text{I}}}^{-}$]: 1 mM, atrazine concentration: 10 mg/L, time: 60 min).

It was mentioned in the previous sections that the reactive species, such as ${{\text{e}}}_{{\text{aq}}}^{-}$,${{\text{SO}}}_{3}^{\cdot -}$ and ${{\text{H}}}^{\cdot }$ are produced during the atrazine degradation by sulfite/iodide/UV process (Eqs. 1 and 2)^20,23^. To clarify the major active species responsible for the atrazine degradation in the sulfite/iodide/UV process, nitrate (${{\text{NO}}}_{3}^{-}$) and nitrite (${{\text{NO}}}_{2}^{-}$) were added to the reaction system as scavengers. (Fig. 11) shows the impacts of ${{\text{NO}}}_{3}^{-}$ and ${{\text{NO}}}_{2}^{-}$ on the degradation of atrazine. Atrazine removal efficiency in the presence of equal amounts of ${{\text{NO}}}_{3}^{-}$ and ${{\text{NO}}}_{2}^{-}$ (10 mM) were about 88% and 91%, respectively. the absence of scavengers, the removal efficiency was 96%, which is due to the scavenging effects of ${{\text{NO}}}_{3}^{-}$ and ${{\text{NO}}}_{2}^{-}$ on ${{\text{e}}}_{{\text{aq}}}^{-}\mathrm{ and }{{\text{H}}}^{\cdot }$, respectively. It was reported that ${{\text{NO}}}_{3}^{-}$ and ${{\text{NO}}}_{2}^{-}$ are good scavengers properties for ${{\text{e}}}_{{\text{aq}}}^{-}$ (Eqs. 13 and 14), but only ${{\text{NO}}}_{2}^{-}$ is capable of quenching ${{\text{H}}}^{\cdot }$ (Eqs. 15 and 16)^18,20,22^. Therefore, according to the inhibitory effect of these two scavengers, the role ${{\text{e}}}_{{\text{aq}}}^{-}$ and ${{\text{H}}}^{\cdot }$ can be determined in the degradation process.

13 $${{\text{NO}}}_{3}^{-}+{\mathrm{ e}}_{{\text{aq}}}^{-}={\left({{\text{NO}}}_{3}^{\cdot }\right)}^{2-} \left({{\text{K}}}_{1 }=9.7\times {10}^{9}{{\text{M}}}^{-1}{{\text{S}}}^{-1}\right),$$

14 $${{\text{NO}}}_{2}^{-}+{\mathrm{ e}}_{{\text{aq}}}^{-}={\left({{\text{NO}}}_{2}^{\cdot }\right)}^{2-}\left({{\text{K}}}_{2 }=4.1\times {10}^{9}{{\text{M}}}^{-1}{{\text{S}}}^{-1}\right),$$

15 $${{\text{NO}}}_{3}^{-}+{{\text{H}}}^{\cdot }={({{\text{NO}}}_{3}{{\text{H}}}^{\cdot })}^{-} \left({{\text{K}}}_{3 }=1.4\times {10}^{6}{{\text{M}}}^{-1}{{\text{S}}}^{-1}\right),$$

16 $${{\text{NO}}}_{2}^{-}+{{\text{H}}}^{\cdot }={{\text{NO}}}^{\cdot }+{{\text{OH}}}^{-} \left({{\text{K}}}_{4 }=7.1\times {10}^{8}{{\text{M}}}^{-1}{{\text{S}}}^{-1}\right).$$

The degradation of atrazine was inhibited by ${{\text{NO}}}_{3}^{-}$ which implies the role of ${{\text{e}}}_{{\text{aq}}}^{-}$ in degrading atrazine. But ${{\text{NO}}}_{3}^{-}$ failed to significantly quench atrazine degradation, suggesting that probably, also sulfite radical is affected atrazine degradation. It is in accordance with the results presented by Cong et al.^23^. They investigated the Cr(VI) removal by sulfite/iodide/UV. results indicated that adding ${{\text{NO}}}_{3}^{-}$ decreased removal efficiency Cr(VI) and ${{\text{e}}}_{{\text{aq}}}^{-}$ has main role in Cr(VI) degradation^23^. Yazdanbakhsh et al. displayed ${{\text{e}}}_{{\text{aq}}}^{-}$ probably plays a minute role in 2, 4, 6-trichlorophenol degradation and ${{\text{SO}}}_{3}^{\cdot -}$ was responsible for degradation via an advanced reduction process based on sulfite anion radical^25^.

To investigate the oxidative species, methanol (75 mM) and ethanol (50 mM) were separately added into the reaction solution as scavengers. Methanol and ethanol would react with ^•^OH while only methanol would react with ${{\text{SO}}}_{4}^{\cdot -}$^18,40,47^. According to (Fig. 11) efficiency removal of atrazine was obtained at 95% and 88% in the presence of methanol and ethanol, respectively. The results indicate that the effect of methanol was insignificant, but ethanol decreased the degradation efficiency of atrazine in sulfite/iodide/UV process. Which means that ^•^OH played a role in the degradation and probably oxidation and reduction occurred together in the process. The pathway of atrazine decomposition in this research also confirms this issue.

### Effects of the presence of anions

**Figure 12:**
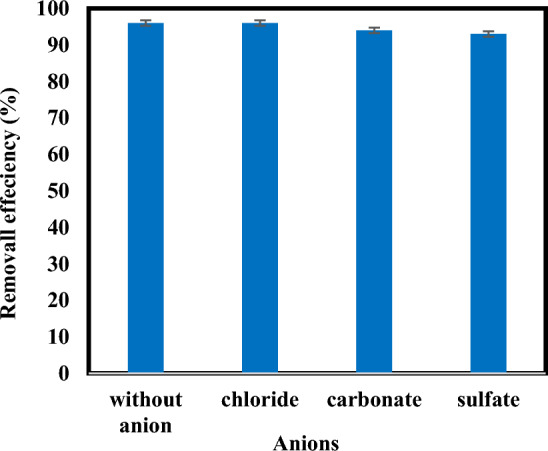
Degradation of atrazine by sulfite/iodide/UV process in presence of anions (pH: 7, [${{\text{SO}}}_{3}^{2-}$] and [${{\text{I}}}^{-}$]: 1 mM, atrazine concentration: 10 mg/L, time: 60 min).

The effect of anions on the performance of advanced reduction processes has been reported in some studies^9,16,23^. So in this study, in addition to nitrate and nitrite anions, the effects of chloride, sulfate, and carbonate anions were investigated. For this purpose, constant amounts of three common inorganic anions including NaCl, Na_2_CO_3_ and Na_2_SO_4_ (10 mM) were added to the solution before starting the reaction under optimum conditions. (Fig. 12) indicates the influence of anions (carbonate, chloride and sulfate) on the removal efficiency of atrazine in sulfite/iodide/UV process. As indicated in (Fig. 12) removal efficiency of atrazine reached 96% in the absence of anions to 96%, 94% and 93% in the presence of ${{\text{Cl}}}^{-}$,${{\text{CO}}}_{3}^{2-}$ and ${{\text{SO}}}_{4}^{2-}$, respectively. It is clear that chloride has no effect on the removal efficiency. Also, the presence of ${{\text{CO}}}_{3}^{2-}$ and ${{\text{SO}}}_{4}^{2-}$ anions has a small effect on reducing the removal efficiency.

The studies show that the negligible influence of anions on the reduction processes in pollutant degradation is due to the following reasons:

1. The minor contribution to ${{\text{e}}}_{{\text{aq}}}^{-}$ in the degradation of the target contaminants^25^,
2. The attraction of UV light by anions. Anions prevent UV rays from interacting with oxidizing and reducing agents in reactions, which in turn reduces the formation of active radicals that degrade the target pollutants^48,49^,
3. The reaction constants and inhibition potential of inorganic ions) chloride, sulfate, and carbonate) show that they are weak inhibitors of ${{\text{e}}}_{{\text{aq}}}^{-} \mathrm{and }{{\text{H}}}^{\cdot }$^16^.

Moussavi et al.^50^ studied the effect of anions in water on the performance of a VUV photoreactor in the removal of acetaminophen. They displayed that sulfate, chloride, carbonate and bicarbonate had no significant impact on the removal efficiency of acetaminophen by the VUV photoreactor which was similar to the results of this study^50^. While, Rasoulzadeh study group (2022) investigated the degradation of Ocuflox in a neutral photo-oxidation/reduction system based on the enhanced heterogeneous-homogeneous sulfite-iodide cycle. Their results showed that the removal efficiency of Ocuflox in UZI and UZS processes decreased in the presence of nitrate, bicarbonate, chloride and sulfate^48^. With these interpretations, the effect of anions on the performance of advanced reduction processes has been complex and depends on reaction conditions, the type of ARP process and the nature of pollutants.

### Mineralization

**Figure 13:**
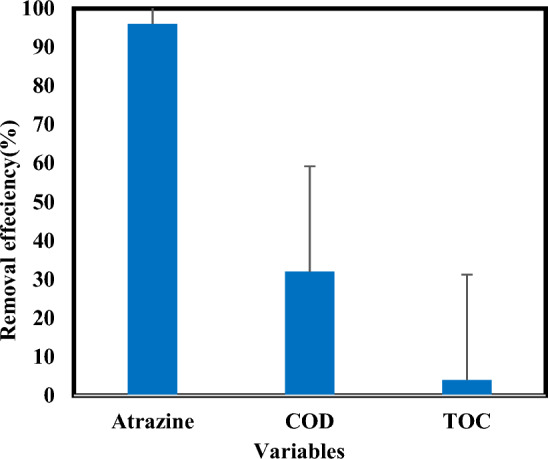
Comparison between mineralization and removal efficiency of atrazine (pH: 7, [${{\text{SO}}}_{3}^{2-}$] and [${{\text{I}}}^{-}$]: 1 mM, atrazine concentration: 10 mg/L, time: 60 min).

The conversion of unstable organic materials to stable inorganic materials is defined as mineralization^48^. The mineralization of atrazine in the sulfite/iodide/UV process was investigated using TOC and COD analysis**.** (Fig. 13) shows that the efficiency of TOC and DOC were 4% and 32%, respectively, while the degradation rate of atrazine under the same experimental conditions was 96%. The low efficiency of atrazine mineralization and the analysis of the atrazine mineralization products and metabolites by LC–MS showed that atrazine was transformed into by-products instead of complete mineralization, which can have intricate and robust molecular structures. As a result, they may exhibit varying reactivity, leading to differences in their removal efficiency based on TOC and COD measurements. Since contact time is an important factor in the mineralization rate, it can also be said that the contact time of 60 min was not enough for the complete mineralization of atrazine and by-products, and probably long reaction times may be needed for the complete mineralization of atrazine^25,51,52^.

Yazdanbakhsh et al. reported that in the dye degradation by dithionite/UV-C advanced reduction process, the mineralization rate was higher with the increased contact time^18^. In research by Mahmoudi et al. for the removal of ofloxacin using the UV/iodide advanced reduction process and biological treatments, it was observed that 30 min after the UV/iodide process, the COD removal efficiency was 40.9%, and when using the bioreactor as a post-treatment at a concentration of MLSS 1000 mg/L, the COD removal rate in 11 h reached 65.28%^53^.

### Intermediates and possible degradation pathways

**Figure 14:**
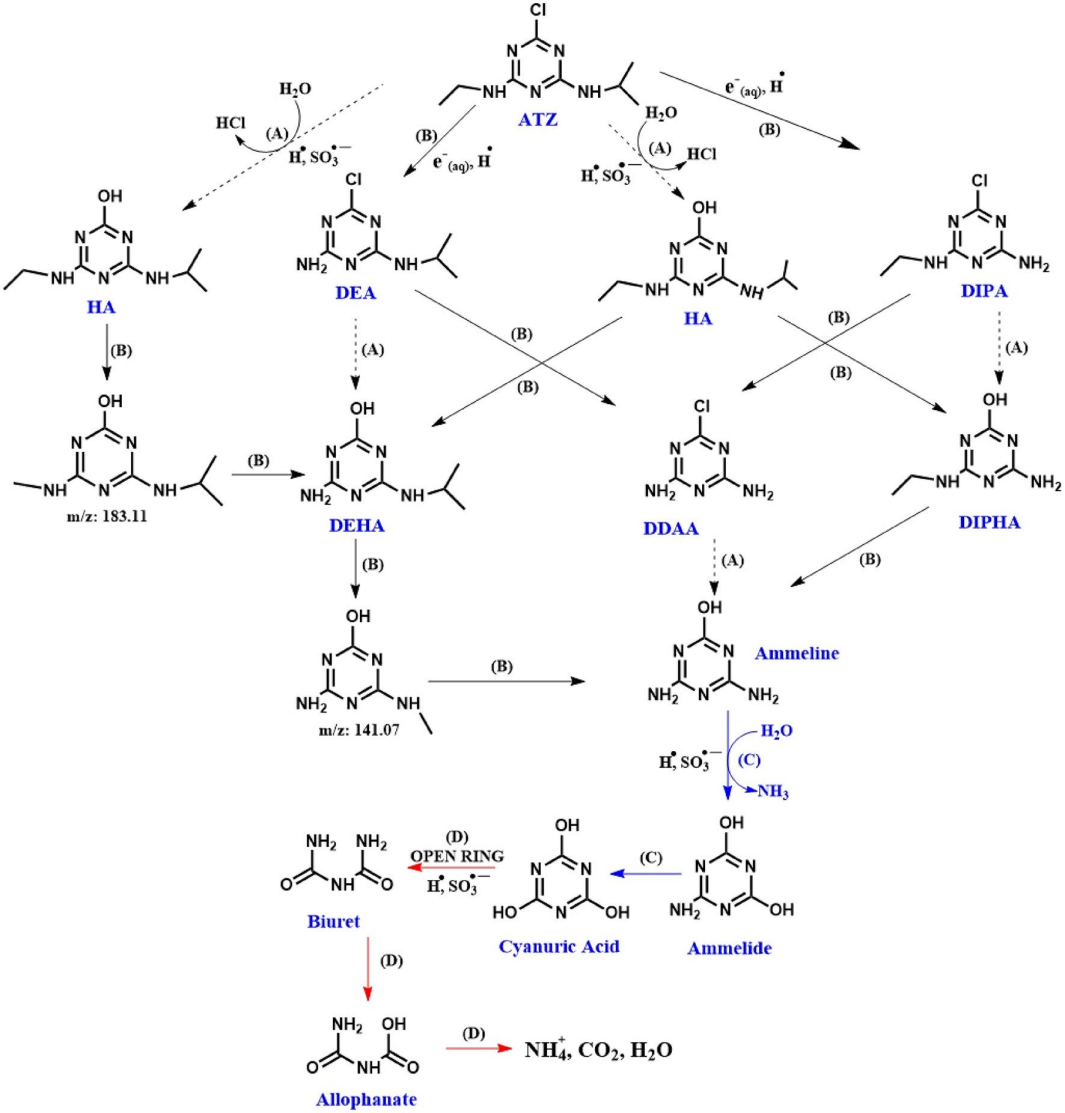
Proposed degradation pathways of atrazine by sulfite/iodide/UV process.

In this study, to identify intermediate species and to clarify the pathway of atrazine decomposition, the by-products resulting from the reaction under optimal conditions were analyzed using the LC–MS technique. Based on the mass spectrum analysis of the atrazine degradation process, intermediate species and proposed structures are shown in Table 3. Among the identified products in the table, no spectrum related to atrazine was observed in the mass spectrum, and the m/z of the identified products is lower than the m/z = 215 of the primary substance. This means that the primary substance has been completely degraded into products with lower m/z. According to the products identified in (Table 3) and the reports of other researchers^11,31,37,54–58^, the proposed mechanism in the study for the degradation of atrazine is reported in (Fig. 14). The LC–MS spectrum of the intermediate produced during the atrazine degradation by sulfite/iodide/UV process has been brought in supplementary (Fig. S1). The general degradation pathways proposed for atrazine include: (A) De-chlorination and hydroxylation of S-triazine ring, (B) De-alkylation of amino groups, (C) Oxidation of amino groups and deamination, and (D) Opening of s-triazine ring. The general degradation pathways proposed for atrazine include path (A) through hydrogen radical and sulfite radical attack and de-chlorination and hydroxylation reactions of the S-triazine ring; path (B) through hydrogen radical attack and hydrated electron and de-alkylation reactions of groups amine; path (C) through the attack of sulfite radical and hydrogen radical and carrying out oxidation reactions of amino groups and deamination; and path (D), which includes the opening of the s-triazine ring through the attack of the active species of hydrogen radical and sulfite.

**Table 3:** LC–MS data for identification of intermediates of atrazine degradation by sulfite/iodide/UV process.

Structure	Name	m/z
	ATZ	216
	HA	197
	DEA	188
	4-(isopropylamino)-6-(methylamino)-1,3,5-triazin-2-ol	183
	DIPA	173
	DIPHA	170
	DEHA	169
	4-amino-6-(methylamino)-1,3,5-triazin-2-ol	141
	DDAA	146
	Ammeline	127
	Ammelide	128
	Cyanuric acid	129
	Biuret	103
	Allophanate	104

The path A includes de-chlorination and hydroxylation of the S-triazine ring related to the atrazine molecule, which leads to the production of the hydroxyl atrazine product (HA). In the continuation of this path, as a result of two successive dimethylation reactions, two products with m/z = 183 and m/z = 169 (Deethylhydroxyatrazine) (DEHA) are produced. Further demethylation of Deethylhydroxy atrazine will produce intermediates with m/z = 141 and ammeline.

The path B path also includes the de-alkylation of amine groups in different positions of the atrazine molecule and the production of Deethylatrazine (DEA) and Deisopropylatrazine (DIPA) intermediates. These two molecules either through dechlorination and hydroxylation of the S-triazine ring lead to the production of intermediates of Deethylatrazine (DEA) and Deisopropyl hydroxyatrazine (DIPHA) or through dealkylation of amine groups, the product (DDAA) creates didealkylatrazine. The continuation of the degradation process of this path, like path A, will also lead to the production on ammeline.

The path C path includes oxidation of amino groups and de-amination of ammeline molecules, which leads to the production of cyanuric acid and amelide products. According to our proposed mechanism and what has been reported in the articles, in all the proposed pathways for the degradation of atrazine, the final products and intermediates resulting from the degradation of atrazine are finally converted into cyanuric acid, amelide and ameline. Because it is very difficult to open and break the S-triazine ring in the early stages of molecule degradation. At the end of all the proposed pathways for the degradation of atrazine, the cyanuric acid product is produced, and in the continuation of the degradation reaction in path D, the S-triazine ring of this molecule is also broken, the ring is opened and the less toxic biuret compound is produced. Biuret is also hydrolyzed to allophanate as shown in the proposed mechanism, followed by final products ${{\text{CO}}}_{2}$, ${{\text{H}}}_{2}$ O and ${{\text{NH}}}_{4}^{+}$ and short-chain acid produced. Therefore, according to this proposed mechanism, mineralization of atrazine has occurred, which reduces the toxicity of the primary compound^11,54–58^.

## Conclusions

The degradation of atrazine by the sulfite/iodide/UV process was investigated. Atrazine removal efficiency of 96% was achieved with an iodide and sulfite concentration of 1 mM and an initial atrazine concentration of 10 mg/L at pH 7. The atrazine removal efficiency increased with increasing atrazine initial concentration in the optimum conditions. The synergy between UV, sulfite and iodide produced ${{\text{SO}}}_{3}^{\cdot -}$ and ${{\text{e}}}_{{\text{aq}}}^{-}$, which significantly contributed to atrazine degradation. The degradation kinetics of atrazine were matched the pseudo-first-order model. The removal efficiency did not change significantly in the presence of anions. According to the removal efficiency of atrazine in the presence of UV, it can be claimed that direct photolysis was also effective in the degradation process in addition to the reaction of atrazine with reducing radicals (${{\text{e}}}_{{\text{aq}}}^{-}$ was a main reducing radical for the degradation process). Based on the LC/MS analysis, no mass spectrum related to atrazine was observed at the end of the reaction time and fourteen byproducts were generated by de-chlorination, hydroxylation, de-alkylation and oxidation reactions that have the capability for conversion to the ${{\text{CO}}}_{2}$ ,${{\text{H}}}_{2}$ O and ${{\text{NH}}}_{4}^{+}$. Overall, sulfite/iodide/UV process illustrates excellent potential for the degradation of atrazine in aqueous solutions.

### Supplementary Information

Supplementary Figure S1.

## Data Availability

All data generated or analyzed during this study are included in this published article and its supplementary information files.
