# Supplementary material for: Photoreduction of atrazine from aqueous solution using sulfite/iodide/UV process, degradation, kinetics and by-products pathway
Source: Sci Rep. 2024 Mar 3;14:5217. doi: 10.1038/s41598-024-55585-6 (PMC10909853; doi:10.1038/s41598-024-55585-6)
Supplement: Supplementary file 1 — Supplementary Figure S1. [file 41598_2024_55585_MOESM1_ESM.docx]

Fig. S1. The LC-Mass spectrum of intermediate produced during the atrazine degradation by sulfite/iodide/UV process.
